# Evolutionary history of the snooks: Phylogeny, biogeography and diversification of the genus *Centropomus*

**DOI:** 10.1371/journal.pone.0332412

**Published:** 2025-10-09

**Authors:** Gabryele Malcher, Thamires Oliveira, Paulo Ronaldo Ferreira, Lucila Melo, Patrícia Mendonça, Omar Dominguez-Dominguez, Arturo Angulo, Claudia Patricia Ornelas-García, Rocío Rodiles-Hernández, Péricles Sena do Rêgo, Juliana Araripe

**Affiliations:** 1 Laboratório de Genética e Conservação, Instituto de Estudos Costeiros (IECOS), Universidade Federal do Pará, Bragança, Pará, Brazil; 2 Laboratorio de Biología Acuática “J. Javier Alvarado Díaz”, Facultad de Biología, Universidad Michoacana de San Nicolás de Hidalgo, Michoacán, Mexico; 3 Instituto Nacional de Biodiversidad, Quito, Ecuador; 4 Escuela de Biología, Museo de Zoología/Centro de Investigación en Biodiversidad y Ecología Tropical (CIBET) and Centro de Investigación en Ciencias del Mar y Limnología (CIMAR), Universidad de Costa Rica, San Pedro de Montes de Oca, San José, Costa Rica; 5 ICtioCR, San Pedro de Montes de Oca, San José, Costa Rica; 6 Colección Nacional de Peces, Instituto de Biología, Universidad Nacional Autónoma de México, Ciudad de Mexico, Mexico; 7 Departamento de Conservación de la Biodiversidad, El Colegio de la Frontera Sur, Chiapas, Mexico; University of Iceland, ICELAND

## Abstract

The snooks (*Centropomus* spp.) are a group of 13 morphologically similar fish species that are widely distributed off the Atlantic and Pacific coasts of the Americas. This study used a multilocus approach to assess the evolutionary relationships within the genus, and to estimate the divergence times of all the taxa. A total of 105 specimens were analyzed throughout the geographic distribution of the different species. The results of the analyses suggest that the genus *Centropomus* is composed of four species groups, which originated in the Miocene (~20 Ma) with the common ancestor of the genus probably inhabiting coastal environments in the Americas prior to its diversification. However, most of the cladogenetic events that determined the extant diversity of the genus occurred more recently, during the Pliocene-Pleistocene transition. The samples analyzed here permitted identify three distinct clades within the *C. parallelus*/*C. mexicanus* complex, with *Centropomus mexicanus* likely being restricted to the Gulf of Mexico. The results of the present study also corroborated the existence of two distinct lineages in *Centropomus parallelus* and identified two previously undescribed lineages in *Centropomus viridis*. The estimates of divergence times indicated that the formation of the Isthmus of Panama played an important factor in the evolution of the snooks, as weel as oscillations in sea level and ecological adaptations. The type of habitat is related to the evolutionary history of the genus, with the ancestral forms likely inhabiting riverine shoreline environments. Our findings highlight the importance of spatially comprehensive sampling for a better understanding of the evolutionary history of the centropomids, and reinforce the need for a more comprehensive taxonomic review of the genus *Centropomus*.

## Introduction

**Fig 1:**
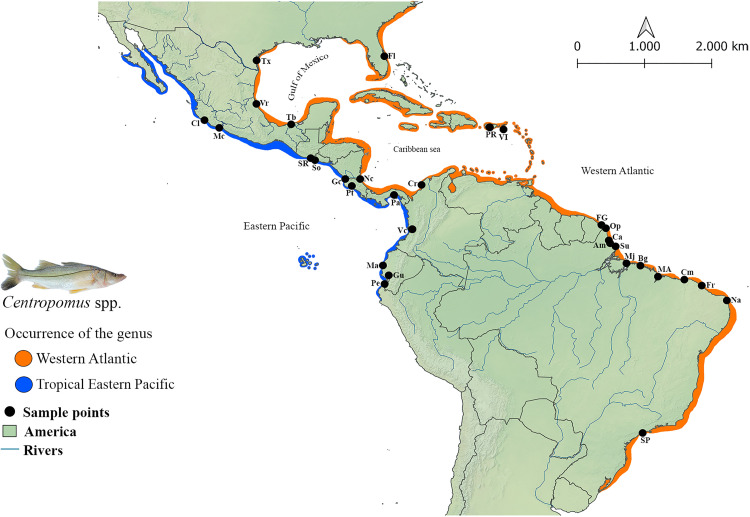
The geographic range of the genus *Centropomus* in the Western Atlantic (orange) and Tropical Eastern Pacific (blue). The black circles represent the sampling points. The codes refer to the collection localities (S1 Table). This map was compiled by the authors using the QGIS software (Geographic Information System – https://www.qgis.org). Adapted from Rivas [4]. *Centropomus irae* obtained by the authors represents the genus.

The fishes of the genus *Centropomus* Lacepède, 1802 (Centropomidae, Carangiformes) [1] are commonly known as snooks, robalos or camurims, respectively, in the English-, Spanish- and Portuguese-speaking regions in which they are found [2,3]. This genus includes 13 species [3], which are found in marine, estuarine, and riverine ecosystems on both the Atlantic and the Pacific coasts of the Americas [2] (Fig 1). Seven of these taxa occur in the Western Atlantic: *Centropomus undecimalis* (Bloch, 1792), *Centropomus parallelus* Poey, 1860, *Centropomus pectinatus* Poey, 1860, *Centropomus ensiferus* Poey, 1860, *Centropomus mexicanus* Bocourt, 1868, *Centropomus poeyi* Chávez, 1961, and the most recently described species *Centropomus irae* Carvalho-Filho, Oliveira, Soares & Araripe, 2019. The remaining species are found in the Tropical Eastern Pacific: *Centropomus armatus* Gill, 1863, *Centropomus nigrescens* Günther, 1864, *Centropomus medius* Günther, 1864, *Centropomus unionensis* Bocourt, 1868, *Centropomus viridis* Lockington, 1877, and *Centropomus robalito* Jordan & Gilbert, 1882.

Most of these taxa have wide distributions, and are either sympatric and/or syntopic in their respective oceans [4]. The species found in the Western Atlantic range between the American state of North Carolina and the Brazilian state of Rio Grande do Sul, including the West Indies [3,5], except for *C. poeyi* and *C. irae*, which have more restricted geographic ranges. While *C. poeyi* is found exclusively in the southwestern Gulf of Mexico, between Aransas Bay, in Texas, United States [6], and Tabasco, in Mexico [4], *C. irae* is endemic to the marine biogeographic region known as the Amazon River Plume (ARP) [5], with all the voucher specimens have been collected off Amapá state, the northernmost state of Brazil [3]. The six *Centropomus* species that occur in the Tropical Eastern Pacific range from southern Baja California, in Mexico, to the northern coast of Peru [4,7]. However, the distribution of *C. viridis* also includes the Galápagos Archipelago, being the only species of the genus recorded on these islands [7]. Thus, this centropomid is the most widely distributed species in the Eastern Pacific [4].

The snooks are stenothermal, euryhaline, diadromous and estuarine-dependent fish [4]. The different species are ecologically diverse [8], inhabiting marine, estuarine, and riverine environments [4,8]. In marine habitats, these fish live in shallow, inshore waters and estuaries [4], with the use of alternative habitats by the adults contributing to niche partitioning among the different species [9]. In some species, such as *C. ensiferus* [8], *C. parallelus* [9], and *C. irae* [3], the adults are found predominantly in riverine coastal areas, whereas in others, such as *C. undecimalis* [10] and *C. mexicanus* [4], the adults are found more commonly in coastal waters of high salinity. Tringali et al. [8] interpreted the interspecific variation in habitat preferences and body size as adaptive differences associated with the evolutionary diversification of the genus *Centropomus*.

The evolutionary relationships within species of the genus *Centropomus* were initially investigated based on the external morphology of the fish [4] and osteological data [11,12]. Subsequently, a number of phylogenetic and taxonomic studies were based on the analysis of molecular data [8,13] or integrated morphological-molecular approaches [14,15]. Despite these efforts, some of the relationships among the *Centropomus* species remain unresolved due to the inconsistencies that persist between the tree topologies revealed in different studies, and the lack of statistical support for some of the branches. For example, Fraser [11] considered *C. armatus* to be closely related to *C. ensiferus*, whereas Tringali et al. [8] and Ossa-Hernandez et al. [13] hypothesized that *C. armatus* is the sister species of *C. unionensis*. Additionally, the studies by Ossa-Hernández and Tringali et al. also recovered the clade formed by *C. pectinatus* and *C. medius* as the sister group of the large clade formed by *C. parallelus*, *C. mexicanus*, *C. nigrescens*, *C. viridis*, *C. poeyi*, *C. undecimalis*, and *C. irae*, although the low nodal support means that this arrangement is still undefined.

In addition to these inconsistencies in some of the evolutionary relationships, a number of taxonomic questions persist, such as the validity of *C. mexicanus* [14]. In fact, the external morphology of this taxon is extremely similar to that of its sister species, *C. parallelus* [4,11,16] and the genetic divergence between these species is also low [8,13]. In this context, Seyoum et al. [17] recently identified three lineages within the “*C. parallelus*/*C. mexicanus* complex”, one lineage being restricted to Florida, and a second in Texas (Gulf of Mexico), while the third lineage is found in Brazil and Puerto Rico. However, the phylogenetic relationships among these lineages, and their taxonomic status, still remain unresolved.

The aim of this study is to investigate the evolutionary history of the genus *Centropomus*. To do this we used a multilocus dataset from all currently recognized species of the genus, with the most extensive spatial sampling of *Centropomus* investigated in phylogenetic studies to date. Here, we infer the phylogenetic relationships within the genus and estimate the timing of divergence among taxa, in order to investigate the drivers of diversification, their biogeographic history, and the factors and scenarios that likely shaped the evolutionary history of the genus.

## Materials and methods

### Ethics statement

All necessary permits were obtained for the described study, which complied with all relevant regulations, and there were no endangered or protected species between the samples. The collection of specimens and the granting of access to genetic material adhered strictly to the legislation of each of the respective countries. In Brazil, the permit was obtained from ICMBio (SISGEN AEE8621), and as follows in Ecuador (013/2012 PNG; N°21–2017-EXP-CM-2016-DNB/MA and MAAE-DBI-CM-2021–0152), El Salvador (MARN-AIMA-004–2013), Mexico (PPF/DGOPA-035/15; CONAPESCA-PPF/DGOPA-262/17; PPF/DGOPA-065/21; PPF/DGOPA-07/23; PPF/DGOPA-025/23; and SAGARPA-CONAPESCA: PPF/ DGOPA249/14), Panama (ARAP PFC-78–2013), and Costa Rica (R-SINAC-SE-DT-PI-029–2023, R-SINAC-ACG-PI-045–2023 and CBio-UCR-R-359).

### Sampling

The present study analyzed the 13 known species of the genus *Centropomus*, a total of 105 individuals, ranging from two of *C. unionensis* to 32 of *C. parallelus* (S1 Table). Tissue samples of 70 specimens were collected during field expeditions, acquired from artisanal fishers, or obtained from scientific collections. These samples were deposited at the *Laboratório de Genética e Conservação* (LGC) of the *Instituto de Estudos Costeiros* (IECOS) of the *Universidade Federal do Pará* (UFPA), in Bragança (Pará), Brazil, and in the scientific collections of the Florida Museum of Natural History (UF, Florida, United States), *Facultad de Biología* of the *Universidad Michoacana de San Nicolas de Hidalgo* (UMSNH, Morelia, México), *Museo de Zoología* of the *Universidad de Costa Rica* (MZUCR, San José, Costa Rica), *Colección Nacional de Peces*, *Instituto de Biología* of the *Universidad Nacional Autónoma de México* (CNPE-IBUNAM, Mexico City, México), and the *Departamento de Conservación de la Biodiversidad* of the *El Colegio de la Frontera Sur* (ECOSC, Chiapas, México).

### Laboratory procedures

The DNA was extracted using the Wizard® Genomic DNA Purification kit (Promega), following the manufacturer’s protocol. Three genes – two mitocondrial and one nuclear – were amplified by Polymerase Chain Reaction (PCR). The mitochondrial rRNA16S and Cytochrome Oxidase subunit I (COI) genes were amplified using the pairs of primers described by Palumbi et al. [18] and Ward et al. [19], respectively. The PCRs were run in a final volume of 13 µl containing 2 µl of dNTPs (1.25 mM), 1.25 µl of 10X PCR buffer (200 mM Tris–HCl, pH 8.4, 500 mM KCl), 1 µl of MgCl_2_ (50 mM), 0.1 µl of *Taq* DNA polymerase (5 U/μl, Invitrogen), 0.5 µl of each primer (50 ng/µl), 1 µl of the DNA (approximately 50 ng/µl), and water to complete the final volume.

The nuclear Receptor-Interacting Serine-Threonine Kinase 4 (RIPK4) gene was amplified by nested PCR, which uses two pairs of primers, one external and other internal, in two independent reactions, as described by Li et al. [20]. The PCR run using the external primers had a final volume of 20 µl, containing 1.6 µl of dNTPs (1.25 mM), 2 µl of 10X PCR buffer (composition as above), 0.8 µl of MgCl_2_ (50 mM), 0.1 µl of Platinum^TM^ *Taq* DNA polymerase (5 U/μl, Invitrogen), 0.8 µl of each primer (50 ng/µl), 0.8 µl of the DNA (approximately 50 ng/µl), and water to complete the final volume. This same reagent composition was used for the internal primers, except for the addition of 1 µl of the PCR product from the first reaction, which required an adjustment in the quantity of water used to complete the final volume.

The PCR protocol conditions and primer sequences used here are presented in S2 Table. After PCR, the samples were subjected to electrophoretic run on a 1.5% agarose gel to confirm amplification and to visualize the intensity and integrity of the band. The PCR products were purified with 20% PEG 8000, using the protocol described by Paithankar & Prasad [21], sequenced using the Big Dye kit (Applied Biosystems), and then injected into an automatic ABI 3500XL sequencer (Applied Biosystems), at the *Instituto de Estudos Costeiros* (IECOS, UFPA, Brazil).

### Molecular analyses

The nucleotide sequences were visualized and inspected in Bioedit v.7.2 [22], aligned using the MUSCLE algorithm [23] in MEGA X [24], and trimmed post-alignment in Bioedit v.7.2 [22] to standardize sequence lengths. After this, the sequences were concatenated in Sequence Matrix v.1.7.8 [25] for the construction of the phylogenetic trees (multilocus and mtDNA), using Bayesian (BI) and Maximum Likelihood (ML) inference methods.

For the BI topologies, the evolutionary models and the data partitioning (per gene or codon) were selected using PartitionFinder v.2.1.1 [26,27], based on the Akaike Information Criterion corrected (AICc) [28,29]. PartitionFinder selected the model GTR + G for all partitions in the multilocus tree, whereas for the mtDNA topology, the best models were GTR + I + G, for the rRNA16S gene and the first and third codon positions of the COI gene, and F81 + I, for the second COI codon position. The BI topologies were inferred using MrBayes v.3.2.7 [30]. The multilocus tree was inferred in two independent runs of 25 million Markov Chain Monte Carlo (MCMC) generations, which were sampled every 2,500 generations, with 10% being discarded as burn-in. The mtDNA tree was also generated in two independent runs of three million MCMC generations, which were sampled every 300 generations, with 10% burn-in. At the end of each run, the parameters obtained from the analyses were summarized, and the convergence of the chains was verified in Tracer v.1.7.2 [31], assuming an Effective Sample Size (ESS) ≥ 200.

The ML topologies were generated in RAxMLGUI 2.0 [32,33], using the GTR evolutionary model as estimated within the software, with the heterogeneity of the nucleotide substitution rates being modeled on the Gamma distribution for all the gene partitions. The ML+thorough bootstrap search algorithm was determined through the application of 10 alternative runs with distinct initial trees. The group support was estimated by 1,000 bootstrap pseudo-replicates, which were used to determine the best ML tree [34].

All the topologies were rooted by an outgroup composed of sequences of representatives of the genera *Lates* Cuvier, 1828 and *Sphyraena* Artedi, 1793 (S1 Table). These genera were considered to be appropriate as the outgroup to *Centropomus*, as shown by the analyses of Li et al. [20] and Girard et al. [1]. These sequences were obtained from the GenBank database at the National Center for Biotechnology Information (NCBI) [35]. The topologies were visualized and edited in FigTree v.1.4.4 [36].

A species tree was generated in order to estimate the Time to the Most Recent Common Ancestor (TMRCA) for the different members of the genus *Centropomus*. This species tree was constructed using the StarBeast3 package [37], run in BEAST v2.7.6 [38]. This approach is based on Bayesian statistical principles and the Multi-Species Coalescent model (MSC), which was used to generate a single species tree from the different gene trees. The HKY + G evolutionary model of nucleotide substitution was used for all three genes individually, based on the strict molecular clock and the Yule Process model as the priors for the phylogenetic tree. Two independent runs of 200 million MCMC generations were implemented, with samples being taken every 20,000 generations, with an initial burn-in of 10% of the chains. The divergence times were calibrated based on substitution rates of 1.2% per million years for the COI gene [39], 1.0% per million years for the rRNA16S gene [8], and the rate estimated for the nuclear gene (RIPK4), using the default settings in BEAST2.

The convergence of the chains was verified in Tracer v.1.7.2 [31], assuming ESS ≥ 200. The log and trees files were combined using LogCombiner v.2.7.6 [40], and the trees were then summarized in TreeAnnotator v.2.7.6 [40], with 10% of the trees generated being discarded as burn-in to produce a maximum credibility (consensus) tree. The consensus tree was visualized and edited in FigTree v.1.4.4 [36].

The biogeographic history of *Centropomus* was reconstructed using the BioGeoBEARS package of the R program [41,42]. The BioGeoBEARS analysis used the dated BEAST phylogeny and the contemporary areas of occurrence of the taxa to model probabilistic biogeographical scenarios to infer ancestral geographic ranges of the lineages. Six models were tested and compared, based on their Log-Likelihood (lnL) and Akaike Information Criterion (AIC) values [41]: the Dispersal-Extinction-Cladogenesis Model (DEC) [43,44], a likelihood version of the parsimony-based Dispersal-Vicariance Analysis model (DIVALIKE) [45], the Bayesian-based BayArea model (BAYAREALIKE) [46], and three versions of these models incorporating the “J” (jump dispersal/founder event speciation) parameter (DEC + J, DIVALIKE+J, and BAYAREALIKE+J) [41,42]. For the best-fitting model, we used Biogeographic Stochastic Mapping (BSM) to summarise and calculate the frequencies of the anagenetic and cladogenetic events [47,48] by taking the mean of event counts. The biogeographic provinces of the Western Atlantic were delineated following Araujo et al. [49] and Tosetto et al. [50], while those of the Tropical Eastern Pacific were based on Robertson & Cramer [51].

Finally, MEGA X program [24] was also used to evaluate the intra- and inter-specific divergence of the *Centropomus* taxa, by generating a genetic divergence matrix based on the Kimura 2 Parameter (K2P) model [52] and calculating standard errors. To place our findings in the context of those reported by Seyoum et al. [17] and to assess the existence and distribution of lineages within the *C. parallelus/ C. mexicanus* complex, we incorporated 16SrRNA and COI gene sequences from lineages available in GenBank (S1 Table) and constructed a haplotype network using the Haploviewer software [53].

## Results

### Evolutionary relationships

**Fig 2:**
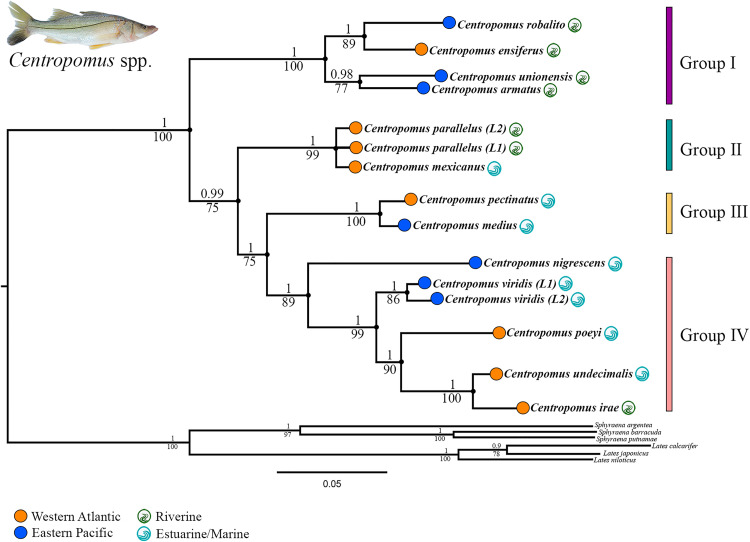
Multilocus topology showing the evolutionary relationships within the genus *Centropomus.* The values shown above the nodes indicate the statistical support defined by the Bayesian Posterior Probability (BPP), while those below the nodes represent the bootstrap (BS) values. *Centropomus irae* obtained by the authors represents the genus.

The concatenated alignment analyzed in the present study has a total of 1602 base pairs (bp), derived from the sequences of the three molecular markers from a total of 83 specimens (S1 Table). The analyses used 78 sequences of the mitochondrial markers, that is, the rRNA16S (454 bp) and COI (578 bp) genes, and 72 sequences of the nuclear RIPK4 gene (570 bp). The multilocus and mtDNA topologies generated here both recovered the same relationships among the species, varying only in their levels of nodal support. Given this, the BI and ML multilocus topologies are presented here (Fig 2) to illustrate the evolutionary relationships within *Centropomus*, while the mtDNA tree is presented in the Supplementary Material (S1A Fig).

The analyses identified the presence of four well-supported species groups within the genus *Centropomus* (Fig 2 and S1A Fig). Group I is composed of four species *C. ensiferus*, *C. robalito*, *C. armatus*, and *C. unionensis*. The monophyly of this group is strongly supported (BPP:1/BS:100) and includes two pairs of sister species: *C. armatus* and *C. unionensis* (BPP:0.98/BS:77), and *C. ensiferus* and *C. robalito* (BPP:1/BS:89).

Group II is formed by the *C. parallelus*/*C. mexicanus* complex (Fig 2 and S1A Fig), with one clade formed by *C. mexicanus* – including all the specimens from the Gulf of Mexico (BPP:0.99/BS:98) – and two other clades that separate the specimens currently recognized as *C. parallelus* into two evolutionary lineages. One (L1) that encompasses the specimens from Central America (Nicaragua) and the other (L2), that includes the specimens from South America (Brazil). These samples form three well-defined mitochondrial haplogroups (S1B Fig). The monophyly of Group II is confirmed by high support values (BPP:1/BS:99), although the polytomy that persists within the group, impedes the determination of which of the two *C. parallelus* lineages is most closely related to *C. mexicanus* (Fig 2).

The topologies recovered here also revealed a close relationship between Group III, formed by *C. medius* and *C. pectinatus*, and Group IV (Fig 2 and S1A Fig). This resolves the relative positions of these taxa. The Group IV is the most diverse of all, and is formed by *C. nigrescens*, *C. viridis*, *C. poeyi*, *C. undecimalis*, and *C. irae*. The *C. viridis* specimens form two evolutionary lineages (Fig 2) in the Tropical Eastern Pacific: one (L1) from Guatemala and Costa Rica, in Central America, and the other (L2) from Ecuador and Peru, in South America (S1A Fig).

Closely-related taxa from the two different oceans – known as trans-isthmian species [4,8,54] – were identified in all groups, except in Group II (Fig 2). In Group I, which was formed by four taxa, *C. ensiferus*, from the Western Atlantic, is closely related to *C. robalito* from the Tropical Eastern Pacific, while in Group III, *C. pectinatus* (Western Atlantic) is a sister species of *C. medius* (Tropical Eastern Pacific). In Group IV, the two *C. viridis* lineages (Tropical Eastern Pacific) are closely related to *C. poeyi*, *C. undecimalis*, and *C. irae,* all from the Western Atlantic.

### Divergence times

**Fig 3:**
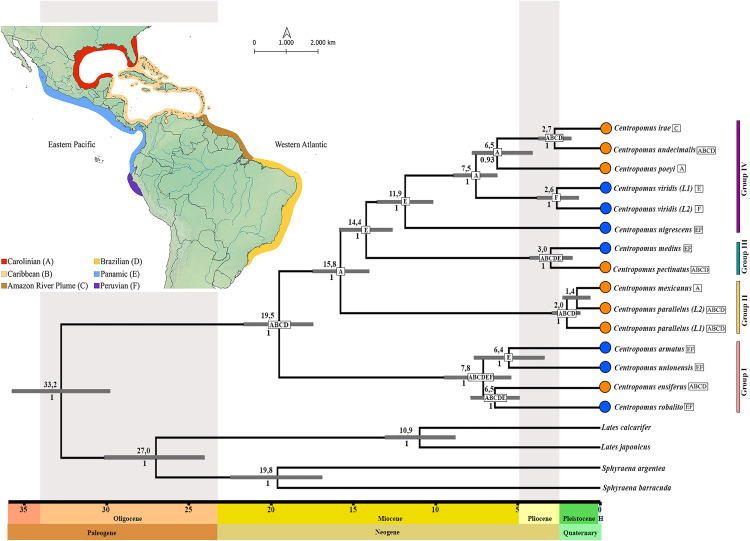
The maximum credibility tree with the Time to the Most Recent Common Ancestor (TMRCA) of the different snook species and the ancestral geographic range of the genus. The gray horizontal bars represent the 95% Highest Posterior Density Interval (HPDi) of the estimated age of each node. The values above the nodes are the mean coalescence times, while those below the nodes represent the statistical support defined by the Bayesian Posterior Probability (BPP). The orange circles represent the taxa from the Western Atlantic and the blue circles, those from the Tropical Eastern Pacific. Node boxes show letters corresponding to the likely ancestral range. The map shows the biogeographical provinces used in the BioGeoBEARS analysis, delineated following Araujo et al. [49] and Tosetto et al. [50] for Western Atlantic, and Robertson & Cramer [51] for Tropical Eastern Pacific. This map was produced by the authors using the QGIS software (https://www.qgis.org).

The species tree with the TMRCA supports the division of the genus *Centropomus* into four species groups, and indicates that it diversified initially at around 19,5 Ma (Highest Posterior Density Interval – HPDi: 17,4–22,6 Ma), that is, in the Early Miocene (Fig 3). The cladogenetic process that resulted in the origin of Group II would likely have occurred during this same geological epoch, at around 15,8 Ma (HPDi: 14,07–17,4 Ma), while the divergence of the ancestors of groups III and IV occurred at approximately 14,4 Ma (HPD:12,6–15,8 Ma).

Within-group diversification began in the Late Miocene (Fig 3). The first of these events occurred in Group IV, at approximately 11,8 Ma (HPDi: 10,1–13,5 Ma), which precedes the diversification within Group I, dated to around 7,8 Ma (HPDi: 5,4–9,4 Ma). The diversification within both groups II and III was more recent, initiating in Group III, at approximately 3,0 Ma (HPDi: 1,6–4,2 Ma) and finally, in Group II, at 2,0 Ma (HPDi: 1,2–2,9 Ma), close to the Plio-Pleistocene transition (Fig 3). The majority of the speciation that occured within the genus *Centropomus* took place during this transition period, between 3,0 Ma and 1,4 Ma, including the divergence of *C. medius* from *C. pectinatus*, *C. irae* from *C. undecimalis*, *C. mexicanus* from the two lineages of *C. parallelus*, and the separation of the two *C. viridis* lineages (Fig 3).

The diversification of the trans-isthmian species was recovered in two temporal events. The first of these events occurred in the Late Miocene, at around 7–6 Ma, with the separation of the trans-isthmian species in both Group I (*C. ensiferus vs*. *C. robalito*), and in Group IV (the ancestor of the *C. viridis* lineages *vs*. *C. poeyi*, *C. undecimalis*, and *C. irae*). Subsequently, in Group III, *C. medius* separated from *C. pectinatus* in the Late Pliocene, at approximately 3 Ma (Fig 3).

### Ancestral geographic range

The DEC + J model had the best fit, based on the LnL and AIC values (S3A Table). The results of the Biogeographic Stochastic Mapping (BSM) indicate that dispersal (58.2%) and founder events (17%) were the principal drivers of speciation in the genus (S3B Table), followed by sympatry (14.5%) and vicariance (10.3%). The BioGeoBEARS analysis indicates that the most probable ancestral area for the genus *Centropomus* during the Early Miocene included the Carolinian, Caribbean, Amazon River Plume, and Brazilian biogeographic provinces (ABCD), all located along the western Atlantic coastline (Fig. 3). Nevertheless, the probability values for ancestral areas at the root were uniformly equal (≤ 2%) across all 63 possible combinations of the six regions analyzed.

The ancestral range recovered for the common ancestor of Group I encompasses coastal environments of both oceans (ABCD+EF), whereas the common ancestor of the *C. parallelus/C. mexicanus* complex (Group II) was restricted to the coastal habitats of the Western Atlantic (ABCD). The ancestral range recovered for *C. pectinatus* and *C. medius* (Group III) includes the coastal environments of the Western Atlantic plus those of the Panamic Province of the Tropical Eastern Pacific (ABCD+E), with subsequent dispersal to the Peruvian Province (F). By contrast, the common ancestor of Group IV was restricted to the coastal environments of the Panamic Province (E) of the Tropical Eastern Pacific. In this group, the ancestral range of the two *C. viridis* lineages was restricted to the coastal habitats of the Peruvian Province (F), followed by a founder event in the Panamic Province (Fig 3).

### Estimates of divergence rates

The genetic divergence matrices for all loci are showed in S4A, S4B and S4C Table. The mean interspecific divergence recorded for the rRNA16S gene ranges from 1.2% in *C. mexicanus vs*. *C. parallelus*_L1 to 17.4% in *C. poeyi vs*. *C. pectinatus*. In the case of the COI gene (Barcode), interspecific divergence ranges from 2.2%, for *C. parallelus*_L2 *vs*. *C. mexicanus*, to 21.5% for *C. unionensis vs*. *C. nigrescens*, whereas the highest divergence value recorded for the nuclear gene (RIPK4) was 3.6%, between *C. mexicanus* and *C. ensiferus*. At the intraspecific level, the greatest divergence in the mitochondrial genes was 0.17% in rRNA16S and 0.43% in COI, both recorded in *C. undecimalis*, whereas in the nuclear RIPK4 gene, intraspecific divergence peaked at 1.78% in *C. unionensis*. The mean interspecific divergence for the Barcode (COI) sequence recorded in the *C. parallelus*/*C. mexicanus* complex was 2.5% between the two *C. parallelus* lineages, 2.7% between *C. parallelus*_L1 and *C. mexicanus*, and 2.2% between *C. parallelus*_L2 and *C. mexicanus*. A Barcode divergence of 3.9% was also found between the two *C. viridis* lineages.

## Discussion

### Evolutionary relationships in the genus *Centropomus*

**Fig 4:**
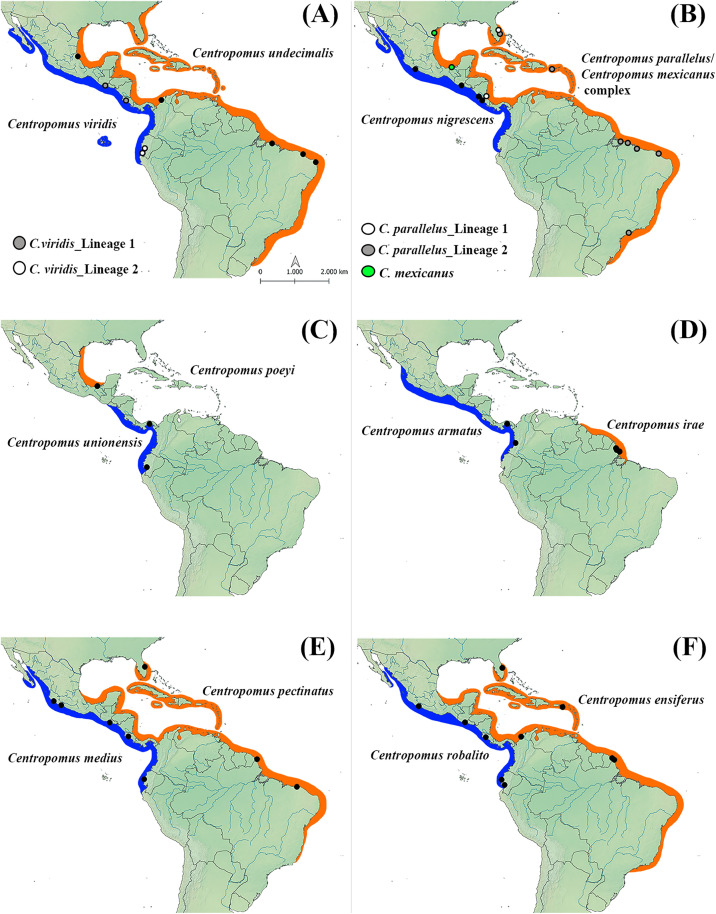
Geographic ranges of the *Centropomus* species found in the Western Atlantic (orange) and Tropical Eastern Pacific (blue). The black circles represent the sampling points. These maps were produced by the authors using the QGIS software (https://www.qgis.org). Adapted from Rivas [4].

The present study provides a comprehensive multilocus assessment of the evolutionary relationships within the genus *Centropomus*, encompassing all species, with samples from different localities across their geographic ranges. In almost all the cases, specimens were obtained from two to five localities, in at least two different countries (Fig 4), with the exception of *C. irae* and *C. poeyi*, which have more restricted ranges, being endemic to the Amazon River Plume and the southwestern Gulf of Mexico, respectively.

This spatial sampling permitted the recognition of lineages that had not previously been identified in the genus, as well as the proposal of hypotheses on the phylogenetic relationships among the different taxa and their respective ancestral ranges. It was also possible to associate the evolutionary history of these fish with geological, oceanographic, paleoclimatic, and ecological parameters. The relationships among the species recovered by the present analyses corroborate the hypotheses of Tringali et al. [8] regarding ecological adaptations of these fish, given that the prefered habitats of the adult fish of the species in the two groups that diverged first (I and II) are the riverine environments of the coastal zone, except in the case of *C. mexicanus* (Fig 2). By contrast, almost all of the species of groups III and IV – with the exception of *C. irae* – tend to be found frequently in coastal zones with higher salinity.

### The small snooks

Four well-supported species groups were recognized within the genus *Centropomus* (Fig 2), which is consistent with the proposal of Tringali et al. [8]. The first of these groups to diverge (Group I) is made up of the small-bodied snooks (*C. ensiferus*, *C. robalito*, *C. armatus*, and *C. unionensis*). The composition of this clade and its monophyly are also supported by the external morphology [4] and osteological parameters [11]. In addition to being the smallest forms of this genus, with a maximum total length of 390 mm [4], the adults of the species of this group also share a preference for riverine coastal habitats [8].

**Fig 5:**
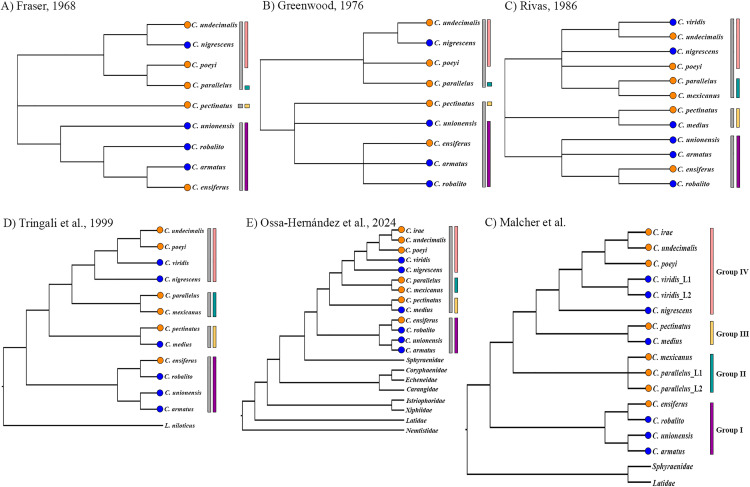
Phylogenetic arrangements proposed for the genus *Centropomus.* Above are the relationships recovered by **(A)** Fraser [11] based on osteological parameters, **(B)** Greenwood [12] based on osteological plus external morphology, and **(C)** Rivas [4] based only external morphology. Below are three distinct molecular phylogenetic arrangements proposed by **(D)** Tringali et al., [8] based on a single locus (rRNA16S), and Ossa-Hernández et al. [13] and Malcher et al. from a multilocus approach. The orange circles represent the taxa from the Western Atlantic and the blue circles, those from the Tropical Eastern Pacific. The gray vertical bars indicate the groups proposed in each previous study and the colored bars indicate the groups proposed in the present study.

The available molecular and morphological analyses have proposed distinct phylogenetic arrangements for the species of this group (Fig 5). In the studies based on external morphology and osteological data, *C. unionensis* was the most divergent of the four species of Group I [4,11,12], while *C. ensiferus* was closely related to either *C. armatus* [11] or *C. robalito* [4]. By contrast, all the phylogenies recovered in the present study placed *C. armatus* as the sister species of *C. unionensis* and confirmed the close relationship between *C. ensiferus* and *C. robalito*, which is consistent with the findings of Tringali et al. [8] and Ossa-Hernandez, et al. [13] (Fig 5). The diversification of this group most likely occurred around 7,8 Ma in the coastal environments of Tropical Eastern Pacific and Western Atlantic (Fig 3).

### The fat snooks (*C. parallelus* and *C. mexicanus*)

One of the most persistent taxonomic questions in the systematics of the genus *Centropomus* is the validity of *C. mexicanus*. Given its major morphological similarities with *C. parallelus* [4,11,14,16] and the low level of molecular divergence between the two taxa [8,14], has led to the proposal of *C. mexicanus* as a junior synonym of *C. parallelus* by Figueiredo-Filho [14]. Morphologically, the two forms can only be differentiated by the scale count along the lateral line and around the caudal peduncle, and they sympatric and syntopic [4]. There are ecological differences, however, given that the adults of *C. parallelus* are found more frequently in riverine coastal environments [9], while those of *C. mexicanus* tend to be found in more saline habitats [4].

Our results revealed three distinct clades within the *C. parallelus/C. mexicanus* complex, corroborating the findings of Seyoum et al. [17]. One of them is exclusively formed by specimens from Tabasco, Mexico, and is supported by a high statistical value (S1A Fig). These specimens share mitochondrial haplotypes with *C. mexicanus* from Texas, USA, previously analyzed by Seyoum et al. [17] (S1B Fig). This results, combined with the genetic divergence values (S4B Table), supports the hypothesis that *C. mexicanus* represents a valid group potentially restricted to the Gulf of Mexico (Fig 4), which is consistent with its type locality [4], contrasting with its proposed synonymy with *C. parallelus* by previous authors, such as Figueiredo-Filho et al. [14]. Notably, all previous molecular studies that indicating a high level of similarity between *C. mexicanus* and *C. parallelus* [8,14] only analyzed sequences from Puerto Rico (see Tringali et al., [8]) or Brazil (see Figueiredo-Filho et al., [14]), and none from the Gulf of Mexico. Therefore, a comprehensive taxonomic revision integrating morphological and molecular data, including species delimitation analyses, should be conducted to accurately resolve species boundaries within this complex.

The phylogenetic reconstruction presented here also revealed that *C. parallelus* is paraphyletic, with two distinct evolutionary lineages (Fig 2), which is consistent with the findings of Seyoum et al. [17]. The samples of this taxon from Florida, Mexico, Nicaragua, and Brazil included in the present study permitted the extension of the known distribution of both lineages. Lineage 1 (L1) ranges between the United States to Central America (Fig 4), with haplotypes being shared between the specimens from Florida and Nicaragua (S1B Fig). The second lineage (L2), in turn, has an ampler distribution, ranging from Florida through the West Indies (Puerto Rico) to the coast of Brazil (Fig 4), with haplotypes being shared between Puerto Rico and Brazil (S1B Fig). This implies that the two *C. parallelus* lineages are sympatric off the Atlantic coast of Florida (Fig 4).

The patterns of genetic divergence (S4A and S4B Tables) recorded within this complex were similar to those recorded between some closely related *Centropomus* taxa, such as *C. undecimalis* and *C. irae* (for the rRNA16S sequences), and are within the range used to differentiate species (2–3%) using the DNA barcode [19,55]. In addition, all the topologies recovered here indicate the presence of three clades within this species complex, with diversification as recent as the Lower Pleistocene, which would be similar to that observed between *C. undecimalis* and *C. irae* (Fig 3).

The *C. parallelus*/*C. mexicanus* complex is denominated Group II here, and is the only group formed exclusively by species from the Western Atlantic. In all the topologies, this group appears as the sister group of the major clade formed by Groups III and IV (Fig 2 and S1A Fig). In the previous molecular proposals (e.g., Tringali et al. [8]; Ossa-Hernandez et al. [13]), *C. parallelus* and *C. mexicanus* appear as the sister group of the species recognized here as Group IV (Fig 5), although with low nodal support. Within Group II, the multilocus and mtDNA topologies obtained using both BI and ML approaches identified a polytomy (Fig 2). Additional genetic data will be needed in future studies to clarify the phylogenetic relationships within this complex. Based on our findings, a comprehensive taxonomic review of this species complex would be recommendable, in order to better elucidate the evolutionary scenario including the relevance of the morphological parameters and the possible description or revalidation of species of fat snook from the Western Atlantic.

### The position of *C. pectinatus* and *C. medius*

*Centropomus pectinatus*, from the Western Atlantic, and *Centropomus medius*, from the Tropical Eastern Pacific, are a classic example of a pair of trans-isthmian geminate species, which have been identified as sister species in the phylogenetic arrangements produced by both molecular [8,13] and morphological data [4] (Fig 5). In these studies, the two species form a sister group within the large clade formed by *C. parallelus*, *C. mexicanus*, *C. nigrescens*, *C. viridis*, *C. poeyi*, *C. undecimalis*, and *C. irae*. However, the reduced statistical support (see Tringali et al. [8]; Ossa-Hernandez, [13]) for this arrangement hampers the conclusive definition of the position of these species within the genus, as discussed above.

All the topologies constructed here identified a close relationship between Groups III and IV, and in particular, the position of *C. pectinatus* and *C. medius* (Group III) as the sister group of *C. nigrescens*, *C. viridis*, *C. poeyi*, *C. undecimalis*, and *C. irae* (Group IV), which together form a major clade. As the adults of both these two groups are found primarily in marine environments [4,8] – except for *C. irae*, which occurs in the Amazon plume [5] – it appears likely that adaptations for more saline coastal environments arose at around 14 Ma.

### Cryptic diversity in *C. viridis*

Our findings also revealed the existence of two reciprocally monophyletic evolutionary lineages within *C. viridis*, with disjunct distributions. One of these lineages (L1) occurs in Central America, while the other (L2) is found in South America (Fig 4). In its current form, *C. viridis* is one of the most widespread centropomids of the Tropical Eastern Pacific, with records ranging from the Gulf of California, in Mexico, to the northern coast of Peru, including the Galápagos Archipelago [8]. The present study is the first to analyze *C. viridis* based on a more extensive geographic sampling, including specimens from Central (Guatemala and Costa Rica) and South America (Ecuador and Peru). This is an important difference in comparison with the previous studies, which were restricted to either Mexico [15] or Panama [8,13].

The results demonstrate that the ancestor of the two *C. viridis* lineages inhabited coastal environments in the Peruvian Province, and underwent a recent process of cladogenesis followed by a founder event in the coastal habitats of the Panamic Province, at around 2,6 Ma (Fig 3). This coincides with the period during which most of the *Centropomus* taxa diverged (e.g., 2,7 Ma for *C. undecimalis* and *C. irae* in Fig 3). The divergence of the mitochondrial sequences between the two lineages (2.2% for 16S rRNA and 3.9% for the DNA barcode in S4A Table) is also consistent with that found in closely-related taxa that have undergone a recent speciation process (such as *C. undecimalis* and *C. irae*). These findings reinforce the need for a taxonomic review of this species, given the probable existence of a new species of white snook in the Tropical Eastern Pacific.

The two *C. viridis* lineages probably diversified at around the time of the formation of the Isthmus of Panama *stricto sensu*, that is, approximately 3 Ma [56]. This geological event changed the pattern of maritime currents in the Eastern Pacific [57,58]. Moreover, the environmental changes in the Late Pleistocene [59], such as a decrease in sea level [60] and shifts in ocean temperatures [61], probably drove the isolation of the populations that originated the two *C. viridis* lineages. Ecological factors, such as habitat preference [8] and local adaptations, may also have contributed to the divergence of the two lineages.

### Drivers of snook diversification

The estimates of the TMRCA of the snooks presented here indicate that the genus *Centropomus* originated in the Early Miocene, probably in the present-day coastal habitats of the Western Atlantic (Fig 3). Ossa-Hernandez et al. [13] hypothesized that the genus arose in the Oligocene in the Tropical Eastern Pacific [13], although they only considered two potential ancestral areas, that is, the Western Atlantic and the Eastern Pacific, which restricted the analytical possibilities. In the present study, by contrast, six biogeographic regions of the Pacific and Atlantic coasts of the Americas were evaluated, which increased considerably the number of possible ancestral areas (to 63 combinations) and, in turn, their relative probabilities. As a result, a number of different areas or combinations of areas had similar probabilities, although the most probable among them was the combination of the four biogeographic provinces of the Western Atlantic (Fig.3).

During the Oligocene and Miocene, the Western Atlantic and Eastern Pacific were connected through the Central American Seaway (CAS) [62] until the complete closure the Isthmus of Panama [56,63], and fossil centropomids from this same epoch have been registered in North America [13]. Given this, it is hypothesized that the common ancestor of the genus *Centropomus* already inhabited riverine coastal environments in the tropical Americas prior to its diversification and the current configuration of these two oceans.

The estimated times of origin and diversification of the genus by Ossa-Hernandez et al. [13], which are earlier than those inferred here, could be associated with the methodologies applied. These authors used node calibrations only based on fossil dating, while the present study employed mutation rates previously established for *Centropomus* [8] and Perciformes [39]. According to our results, the origin and diversification of the genus occurred in the Early Miocene. During this geological epoch, the slow and gradual formation of the Isthmus of Panama was initiated through tectonic events with the progressive closure of the maritime connection between the Pacific and Atlantic oceans [56,64]. At approximately 25 Ma [64,65], the chain of semi-emergent islands of the Panamanian Arc began to collide with South America, with the deep-water connection between the oceans being interrupted at some time between 12 Ma and 7 Ma [63,66,67]. Even so, shallow-water connections persisted [56,62], e.g., through the Panama Canal basin [64]. The complete interruption of the flow of water between the Western Atlantic and Eastern Pacific only occurred at approximately 3 Ma [35,40] with the formation of the Isthmus of Panama *stricto sensu*.

The formation of the land bridge that connects North and South America was the vicariant event that most influenced the speciation processes of the sister taxa that currently inhabit the Western Atlantic and Eastern Pacific [56], such as those of the fish family Scianidae [68], which has a very similar distribution and ecological traits to the Centropomidae. This is also valid for the snooks, given that the evolutionary history of the trans-isthmian pairs of *Centropomus* species coincided with this period, beginning in the Miocene (~ 12 Ma) and culminating during the Plio-Pleistocene transition (~ 3 Ma).

One other factor that may have had a significant influence on the evolution of the genus *Centropomus* is the oscillations in sea level that occurred during the Plio-Pleistocene transition [60], which were caused by the successive glacial cycles [59,69]. This is supported by the relatively recent speciation of the snooks, given that nine of the 13 current taxa arose during the past 3,0 Ma to 1,4 Ma (Fig 3). Fluctuations in sea level also had profound impacts on the speciation process in a number of other groups of marine fishes [49,70]. These fluctuations resulted in the reduction of coastal habitats and dynamic shifts in the temperature and salinity of the water column [60], which greatly increased the potential formation of biogeographic barriers [49] and contributed to the isolation of snook populations. The speciation of *C. undecimalis* and *C. irae*, for example, appears to have been intimately related to the influence of the plume of freshwater which emanates from the mouth of the Amazon River, during the periods of low sea level that occurred during the Pleistocene [5].

Despite the known ecological plasticity of *Centropomus*, the adults of each species tend to prefer specific types of habitats [8–10]. According to Tringali et al. [8], the evolutionary trend in this genus ranges from riverine habitats to marine environments, a hypothesis corroborated by the topologies presented here (Fig. 2). Our results indicate that the first groups to diverge (Group I and II) are formed by species with a preference for riverine coastal habitats, except for *C. mexicanus*. The preference for marine environments in snooks probably began around 14,2 Ma, with the ancestor of Groups III and IV. Given this, all of the taxa derived from this process are found predominantly in more saline coastal environments, except for *C. irae*. These traits would thus have arisen independently during the evolution of the centropomids in two recent events: with *C. mexicanus* invading marine coastline environments at around 1,4 Ma, and *C. irae* regressing into riverine habitats at approximately 2,7 Ma. Overall, then, the findings of the present study indicate that ecological factors and the local adaptation of species to specific types of habitats were important drivers of the diversification process in the genus *Centropomus*.

## Conclusions

The present study provides a number of important insights into the evolutionary history of the genus *Centropomus*, a morphologically homogeneous group of fish that is widely distributed on the Pacific and Atlantic coasts of the Americas. The multilocus approach used here determined that genus originated in the Miocene, at around 20 Ma. The common ancestor of the centropomid species probably already inhabited riverine coastal environments in the Americas prior to its diversification. In addition, the genus *Centropomus* was divided into four well-defined species groups, with the majority of the species diversifying more recently, at around the Plio-Pleistocene transition.

The geographic coverage of the present study permitted identify three distinct clades within the *C. parallelus*/*C. mexicanus* complex, supporting the hypothesis that *C. parallelus* and *C. mexicanus* are distinct, with the latter probably restricted to the Gulf of Mexico. The data also corroborated the existence of two *C. parallelus* lineages, which are sympatric off the Atlantic coast of Florida. Two *C. viridis* lineages were also identified, but in this case, with an allopatric distribution.

The formation of the Isthmus of Panama played an important role in the speciation of *Centropomus*, together with oscillations in sea level. Adaptations linked to ecological factors, such as the habitat preferences of adult fish, also appeared to play an important role in this evolutionary process, with the more ancient ancestors of the genus apparently being adapted to riverine coastal environments. Two independent habitat invasion processes were also identified, with *C. mexicanus* invading more saline environments at around 1,4 Ma, and *C. irae* regressing to riverine coastal habitats at approximately 2,7 Ma.

The results of the present study highlight the importance of a comprehensive approach based on broad geographic sampling, which was fundamental to the understanding of the evolutionary history of the study group. Sampling across different localities for each taxon revealed the existence of independent evolutionary lineages, and provided an important contribution to the understanding of the phylogeny of the genus *Centropomus*. This approach is especially relevant for the analysis of the evolutionary history of organisms with an ample distribution and highly conserved external morphology, such as the snooks [4]. The lineages identified here emphasize the need for further research, including a taxonomic review and the application of species delimitation methods, to provide a more information of the diversity of the snooks in the Western Atlantic and Tropical Eastern Pacific.

## Supporting information

S1 Table The *Centropomus* samples (per species) in the present study. Samples points (p); Locality; Accession number of the sequence from the public database (Genbank); Collection: *Laboratório de Genética e Conservação* of the *Universidade Federal do Pará* (LGC/UFPA), Florida Museum of Natural History (UF), *Museo de Zoología* of the *Universidad de Costa Rica* (MZUCR), *Colección Nacional de Peces* of the *Universidad Nacional Autónoma de México* (CNPE-IBUNAM), *Universidade Michoacana de San Nicolás de Hidalgo* (UMSNH), and the *Departamento de Conservación de la Biodiversidad* of the El Colegio de la Frontera Sur (ECOSUR). In brackets, the identification number of the samples in the collections. The asterisk (*) indicates sequences used only in the haplotype network of the *C. parallelus*/*C. mexicanus* complex.
(CSV)

S2 Table Description of the primers and PCR protocol conditions used in the present study. ^1^External primers; ^2^ Internal primers.
(DOCX)

S3 Table Result of the BioGeoBEARS analysis. A) The parameters and scores derived from each BioGeoBEARS model used in the analyses. Values of log-likelihood (LnL); number of estimated parameters (np): rate of dispersal (d), extinction (e), and founder event/jump dispersal (j); Akaike information criterion (AIC). The best model is highlighted in gray. B) Summary of Biogeographical Stochastic Mapping (BSM) counts using the DEC + j model. Mean numbers of the different types of events estimated (Me); founder event/jump dispersal (j); range-switching (a); dispersal (d); extinction (e); vicariance (v); simpatry (y); percentage (%).
(DOCX)

S4 Table Mean intra- and inter-specific pairwise genetic divergence recorded in the present study. Matrices for rRNA16S (A), COI (B), and for the nuclear gene RIPK4 (C); nl = null; n/c = not calculated. Standard deviation values are indicated in gray.
(DOCX)

S1 Fig A) The mtDNA tree showing the relationships among the taxa of the genus *Centropomus.* B) the mitochondrial haplotype network of the *C. parallelus*/*C. mexicanus* complex. In the mtDNA topology, the values shown in the nodes indicate the statistical support defined by the Bayesian Posterior Probability (BPP) and Bootstrap (BS) values, respectively. *Centropomus irae* obtained by the authors represents the genus. In the haplotype network the scale (size of the circle) is proportional to the number of individuals with the respective haplotype.
(TIF)
