# Supplementary material for: Evolutionary history of the snooks: Phylogeny, biogeography and diversification of the genus Centropomus
Source: PLoS One. 2025 Oct 9;20(10):e0332412. doi: 10.1371/journal.pone.0332412 (PMC12510552; doi:10.1371/journal.pone.0332412)
Supplement: S2 Table — 1External primers; 2 Internal primers. (DOCX) [file pone.0332412.s002.docx]

| Gene | Primers | PCR protocol conditions |
| --- | --- | --- |
| rRNA16S | L1987: 5’ GCCTCGCCTGTTTACCAAAAAC 3’  H2609: 5’ CCGGTCTGAACTCAGATCACGT 3’ | Initial denaturation at 94°C for 3’, followed by 35 cycles of 94°C for 45’’, 56°C for 45’’, and 72°C for 2’, with a final extension at 72°C for 5’ |
| COI | Fish_F2: 5' TCGACTAATCATAAAGATATCGGCAC 3'  Fish_R2: 5' ACTTCAGGGTGACCGAAGAATCAGAA 3' | Initial denaturation at 94°C for 7’, followed by 35 cycles of 95°C for 30’’, 55°C for 1’, and 72°C for 2’, with a final extension at 72°C for 10’ |
| RIPK4 | ^1^F57: 5' GCCAAGTTGATGAAGATCCTVCAG 3'  ^1^R880: 5' CCCTCTTCTATCAGCATYTTRACTGT 3' | Initial denaturation at 95°C for 30’’, followed by 15 cycles of 98°C for 10’’, 60°C for 30’’, and 72°C for 45’’, then 15 cycles of 98°C for 10’’, 58°C for 30’’, and 72°C for 45’’, with a final extension at 72°C for 5’ |
|  | ^2^F65: 5' GATGAAGATCCTVCAGCCTCA 3'  ^2^R766: 5' AGACGAGARGTGCTGGTGTG 3' | Initial denaturation at 95°C for 30’’, followed by 15 cycles of 98°C for 10’’, 58°C for 30’’, and 72°C for 45’’, then 15 cycles of 98°C for 10’’, 56°C for 30’’, and 72°C for 45’’, with a final extension at 72°C for 5’ |
