# Supplementary material for: Evolutionary history of the snooks: Phylogeny, biogeography and diversification of the genus Centropomus
Source: PLoS One. 2025 Oct 9;20(10):e0332412. doi: 10.1371/journal.pone.0332412 (PMC12510552; doi:10.1371/journal.pone.0332412)
Supplement: S3 Table — A) The parameters and scores derived from each BioGeoBEARS model used in the analyses. Values of log-likelihood (LnL); number of estimated parameters (np): rate of dispersal (d), extinction (e), and founder event/jump dispersal (j); Akaike information criterion (AIC). The best model is highlighted in gray. B) Summary of Biogeographical Stochastic Mapping (BSM) counts using the DEC + j model. Mean numbers of the different types of events estimated (Me); founder event/jump dispersal (j); range-switching (a); dispersal (d); extinction (e); vicariance (v); simpatry (y); percentage (%). (DOCX) [file pone.0332412.s003.docx]

| **Models** | **LnL** | **np** | **d** | **e** | **j** | AIC |
| --- | --- | --- | --- | --- | --- | --- |
| DEC | -56.3890277913316 | 2 | 0.0261317557125637 | 1e-12 | 0 | 116.8 |
| DEC+J | -54.1695965560943 | 3 | 0.0231850021852143 | 1e-12 | 0.406597969363828 | 114.3 |
| DIVALIKE | -56.567586593671 | 2 | 0.0310337024131548 | 1e-12 | 0 | 117.1 |
| DIVALIKE+J | -54.5944676224418 | 3 | 0.0234310358935345 | 1e-12 | 0.88542143180943 | 115.2 |
| BAYAREALIKE | -77.9919520136092 | 2 | 1.37953713461686 | 1.83841334344668 | 0 | 160.0 |
| BAYAREALIKE+J | -56.5010498787797 | 3 | 0.0188009090102535 | 1e-07 | 0.675744393845066 | 119.0 |

A)

B)

|  | *j* | *a* | *d* | *e* | *v* | *y* | Total |
| --- | --- | --- | --- | --- | --- | --- | --- |
| Me | 5.69 | 0 | 19.46 | 0 | 3.44 | 4.85 | 33.46 |
| % | 17.0 | 0 | 58.2 | 0 | 10.3 | 14.5 |  |
