# Supplementary material for: Evolutionary history of the snooks: Phylogeny, biogeography and diversification of the genus Centropomus
Source: PLoS One. 2025 Oct 9;20(10):e0332412. doi: 10.1371/journal.pone.0332412 (PMC12510552; doi:10.1371/journal.pone.0332412)
Supplement: S4 Table — Matrices for rRNA16S (A), COI (B), and for the nuclear gene RIPK4 (C); nl = null; n/c = not calculated. Standard deviation values are indicated in gray. (DOCX) [file pone.0332412.s004.docx]

|  | **1** | **2** | **3** | **4** | **5** | **6** | **7** | **8** | **9** | **10** | **11** | **12** | **13** | **14** | **15** |
| --- | --- | --- | --- | --- | --- | --- | --- | --- | --- | --- | --- | --- | --- | --- | --- |
| **1. *C. robalito*** |  | 0.009 | 0.015 | 0.012 | 0.017 | 0.017 | 0.017 | 0.021 | 0.020 | 0.021 | 0.020 | 0.019 | 0.022 | 0.020 | 0.021 |
| **2. *C. ensiferus*** | 0.037 |  | 0.014 | 0.010 | 0.017 | 0.017 | 0.017 | 0.020 | 0.019 | 0.020 | 0.019 | 0.019 | 0.021 | 0.020 | 0.021 |
| **3. *C. unionensis*** | 0.078 | 0.072 |  | 0.013 | 0.018 | 0.017 | 0.019 | 0.020 | 0.019 | 0.021 | 0.017 | 0.016 | 0.019 | 0.019 | 0.019 |
| **4. *C. armatus*** | 0.054 | 0.038 | 0.060 |  | 0.018 | 0.018 | 0.018 | 0.018 | 0.017 | 0.021 | 0.019 | 0.019 | 0.022 | 0.021 | 0.022 |
| **5. *C. parallelus_*L1** | 0.111 | 0.111 | 0.123 | 0.114 |  | 0.006 | 0.005 | 0.018 | 0.017 | 0.020 | 0.017 | 0.016 | 0.017 | 0.018 | 0.019 |
| **6. *C. parallelus_*L2** | 0.108 | 0.106 | 0.119 | 0.117 | 0.014 |  | 0.006 | 0.018 | 0.016 | 0.020 | 0.017 | 0.015 | 0.018 | 0.017 | 0.018 |
| **7. *C. mexicanus*** | 0.114 | 0.111 | 0.131 | 0.117 | 0.012 | 0.017 |  | 0.019 | 0.017 | 0.020 | 0.018 | 0.017 | 0.018 | 0.018 | 0.019 |
| **8. *C. pectinatus*** | 0.150 | 0.145 | 0.136 | 0.119 | 0.114 | 0.117 | 0.123 |  | 0.008 | 0.021 | 0.020 | 0.019 | 0.023 | 0.023 | 0.023 |
| **9*. C. medius*** | 0.141 | 0.126 | 0.120 | 0.109 | 0.103 | 0.103 | 0.109 | 0.025 |  | 0.022 | 0.020 | 0.019 | 0.023 | 0.022 | 0.022 |
| **10. *C. nigrescens*** | 0.161 | 0.150 | 0.162 | 0.156 | 0.142 | 0.141 | 0.142 | 0.161 | 0.163 |  | 0.018 | 0.017 | 0.019 | 0.019 | 0.021 |
| **11. *C. viridis_*L2** | 0.143 | 0.132 | 0.108 | 0.134 | 0.111 | 0.108 | 0.117 | 0.139 | 0.140 | 0.120 |  | 0.007 | 0.012 | 0.011 | 0.012 |
| **12*. C. viridis_*L1** | 0.137 | 0.133 | 0.102 | 0.134 | 0.100 | 0.097 | 0.105 | 0.124 | 0.125 | 0.118 | 0.022 |  | 0.013 | 0.013 | 0.013 |
| **13*. C. poeyi*** | 0.162 | 0.154 | 0.134 | 0.168 | 0.117 | 0.125 | 0.123 | 0.174 | 0.169 | 0.136 | 0.057 | 0.062 |  | 0.012 | 0.015 |
| **14*. C. undecimalis*** | 0.139 | 0.142 | 0.124 | 0.152 | 0.117 | 0.117 | 0.120 | 0.169 | 0.154 | 0.133 | 0.051 | 0.062 | 0.062 |  | 0.006 |
| **15*. C. irae*** | 0.156 | 0.158 | 0.127 | 0.163 | 0.128 | 0.125 | 0.131 | 0.173 | 0.158 | 0.152 | 0.059 | 0.065 | 0.078 | 0.018 |  |
| **Intraspecific rRNA16S** | nl | 0.0013 | nl | nl | nl | nl | nl | 0.0015 | nl | nl | n/c | nl | nl | 0.0017 | nl |
|  | - | 0.0009 | - | - | - | - | - | 0.0015 | - | - | - | - |  | 0.0012 | - |

|  | **1** | **2** | **3** | **4** | **5** | **6** | **7** | **8** | **9** | **10** | **11** | **12** | **13** | **14** | **15** |
| --- | --- | --- | --- | --- | --- | --- | --- | --- | --- | --- | --- | --- | --- | --- | --- |
| 1. ***C._robalito*** |  | 0.016 | 0.018 | 0.018 | 0.020 | 0.019 | 0.019 | 0.021 | 0.021 | 0.020 | 0.022 | 0.022 | 0.022 | 0.020 | 0.020 |
| 1. ***C. ensiferus*** | 0.111 |  | 0.017 | 0.016 | 0.021 | 0.021 | 0.022 | 0.020 | 0.022 | 0.021 | 0.022 | 0.022 | 0.021 | 0.021 | 0.021 |
| 1. ***C. unionensis*** | 0.145 | 0.140 |  | 0.015 | 0.023 | 0.022 | 0.022 | 0.021 | 0.021 | 0.023 | 0.022 | 0.021 | 0.021 | 0.021 | 0.020 |
| 1. ***C. armatus*** | 0.147 | 0.123 | 0.101 |  | 0.020 | 0.021 | 0.021 | 0.021 | 0.021 | 0.020 | 0.021 | 0.020 | 0.021 | 0.021 | 0.021 |
| 1. ***C. parallelus_L1*** | 0.171 | 0.191 | 0.209 | 0.180 |  | 0.007 | 0.007 | 0.018 | 0.019 | 0.018 | 0.019 | 0.017 | 0.020 | 0.019 | 0.020 |
| 1. ***C. parallelus_L2*** | 0.161 | 0.186 | 0.205 | 0.183 | 0.025 |  | 0.006 | 0.018 | 0.019 | 0.018 | 0.017 | 0.017 | 0.020 | 0.018 | 0.018 |
| 1. ***C. mexicanus*** | 0.168 | 0.193 | 0.205 | 0.184 | 0.027 | 0.022 |  | 0.018 | 0.018 | 0.018 | 0.018 | 0.018 | 0.020 | 0.018 | 0.019 |
| 1. ***C. pectinatus*** | 0.194 | 0.183 | 0.197 | 0.184 | 0.145 | 0.142 | 0.145 |  | 0.009 | 0.018 | 0.016 | 0.016 | 0.018 | 0.018 | 0.019 |
| 1. ***C. medius*** | 0.191 | 0.200 | 0.192 | 0.188 | 0.152 | 0.151 | 0.143 | 0.048 |  | 0.019 | 0.017 | 0.017 | 0.018 | 0.019 | 0.020 |
| 1. ***C. nigrescens*** | 0.183 | 0.192 | 0.215 | 0.181 | 0.144 | 0.141 | 0.142 | 0.151 | 0.157 |  | 0.019 | 0.019 | 0.017 | 0.017 | 0.019 |
| 1. ***C. viridis_L2*** | 0.209 | 0.195 | 0.201 | 0.183 | 0.155 | 0.144 | 0.151 | 0.123 | 0.133 | 0.162 |  | 0.008 | 0.016 | 0.014 | 0.015 |
| 1. ***C. viridis_L1*** | 0.209 | 0.207 | 0.191 | 0.174 | 0.138 | 0.136 | 0.145 | 0.120 | 0.132 | 0.157 | 0.039 |  | 0.015 | 0.014 | 0.015 |
| 1. ***C. poeyi*** | 0.205 | 0.196 | 0.200 | 0.198 | 0.182 | 0.176 | 0.172 | 0.154 | 0.154 | 0.147 | 0.120 | 0.116 |  | 0.016 | 0.015 |
| 1. ***C. undecimalis*** | 0.194 | 0.202 | 0.198 | 0.199 | 0.172 | 0.156 | 0.165 | 0.157 | 0.168 | 0.147 | 0.104 | 0.110 | 0.135 |  | 0.011 |
| 1. ***C. irae*** | 0.185 | 0.199 | 0.188 | 0.197 | 0.179 | 0.160 | 0.170 | 0.170 | 0.177 | 0.157 | 0.112 | 0.118 | 0.115 | 0.062 |  |
| **Intraspecific COI** | 0.0011 | 0.0015 | 0.0034 | nl | 0.0017 | 0.0099 | 0.0008 | 0.0023 | 0.0014 | 0.0028 | nl | 0.0023 | 0.0008 | 0.0043 | 0.0010 |
|  | 0.0011 | 0.0008 | 0.0024 | - | 0.0012 | 0.0006 | 0.0008 | 0.0016 | 0.0010 | 0.0013 | - | 0.0016 | 0.0008 | 0.0021 | 0.0010 |

|  | **1** | **2** | **3** | **4** | **5** | **6** | **7** | **8** | **9** | **10** | **11** | **12** | **13** | **14** |
| --- | --- | --- | --- | --- | --- | --- | --- | --- | --- | --- | --- | --- | --- | --- |
| 1. ***C.robalito*** |  | 0.005 | 0.003 | 0.007 | 0.007 | 0.008 | 0.007 | 0.007 | 0.008 | 0.008 | 0.007 | 0.007 | 0.008 | 0.008 |
| 1. ***C. ensiferus*** | 0.016 |  | 0.005 | 0.007 | 0.007 | 0.008 | 0.007 | 0.007 | 0.008 | 0.008 | 0.007 | 0.007 | 0.007 | 0.007 |
| 1. ***C.unionensis*** | 0.008 | 0.017 |  | 0.007 | 0.006 | 0.007 | 0.006 | 0.006 | 0.007 | 0.007 | 0.007 | 0.007 | 0.007 | 0.007 |
| 1. ***C. parallelus_L1*** | 0.031 | 0.032 | 0.029 |  | 0.000 | 0.001 | 0.004 | 0.004 | 0.005 | 0.005 | 0.005 | 0.005 | 0.005 | 0.005 |
| 1. ***C.parallelus_L2*** | 0.031 | 0.031 | 0.029 | 0.001 |  | 0.001 | 0.004 | 0.004 | 0.005 | 0.005 | 0.005 | 0.005 | 0.005 | 0.005 |
| 1. ***C.mexicanus*** | 0.035 | 0.036 | 0.033 | 0.003 | 0.003 |  | 0.005 | 0.005 | 0.006 | 0.006 | 0.005 | 0.005 | 0.006 | 0.006 |
| 1. ***C.pectinatus*** | 0.028 | 0.028 | 0.027 | 0.011 | 0.011 | 0.015 |  | 0.001 | 0.004 | 0.004 | 0.004 | 0.004 | 0.004 | 0.004 |
| 1. ***C.medius*** | 0.028 | 0.028 | 0.028 | 0.010 | 0.010 | 0.014 | 0.001 |  | 0.004 | 0.004 | 0.004 | 0.003 | 0.004 | 0.004 |
| 1. ***C. nigrescens*** | 0.032 | 0.032 | 0.032 | 0.014 | 0.013 | 0.018 | 0.008 | 0.007 |  | 0.003 | 0.003 | 0.002 | 0.003 | 0.004 |
| 1. ***C. viridis_L2*** | 0.034 | 0.034 | 0.034 | 0.016 | 0.015 | 0.019 | 0.010 | 0.009 | 0.005 |  | 0.000 | 0.002 | 0.003 | 0.004 |
| 1. ***C. viridis_L1*** | 0.032 | 0.032 | 0.032 | 0.014 | 0.013 | 0.018 | 0.008 | 0.007 | 0.005 | 0.000 |  | 0.002 | 0.003 | 0.004 |
| 1. ***C. poeyi*** | 0.032 | 0.032 | 0.032 | 0.014 | 0.014 | 0.018 | 0.009 | 0.008 | 0.004 | 0.003 | 0.003 |  | 0.002 | 0.003 |
| 1. ***C. undecimalis*** | 0.034 | 0.030 | 0.033 | 0.016 | 0.015 | 0.019 | 0.010 | 0.009 | 0.005 | 0.007 | 0.007 | 0.004 |  | 0.002 |
| 1. ***C. irae*** | 0.035 | 0.031 | 0.034 | 0.017 | 0.017 | 0.021 | 0.012 | 0.011 | 0.007 | 0.009 | 0.009 | 0.006 | 0.002 |  |
| **Intraspecific RIPK4** | 0.0008 | 0.0006 | 0.017 | nl | 0.006 | 0.001 | 0.0011 | nl | nl | n/c | nl | 0.002 | nl | nl |
|  | 0.0007 | 0.0004 | 0.005 | - | 0.004 | 0.001 | 0.0008 | - | - | - | - | 0.001 | - | - |
