# Supplementary figures and images for: Evolutionary history of the snooks: Phylogeny, biogeography and diversification of the genus Centropomus
Source: PLoS One. 2025 Oct 9;20(10):e0332412. doi: 10.1371/journal.pone.0332412 (PMC12510552; doi:10.1371/journal.pone.0332412)

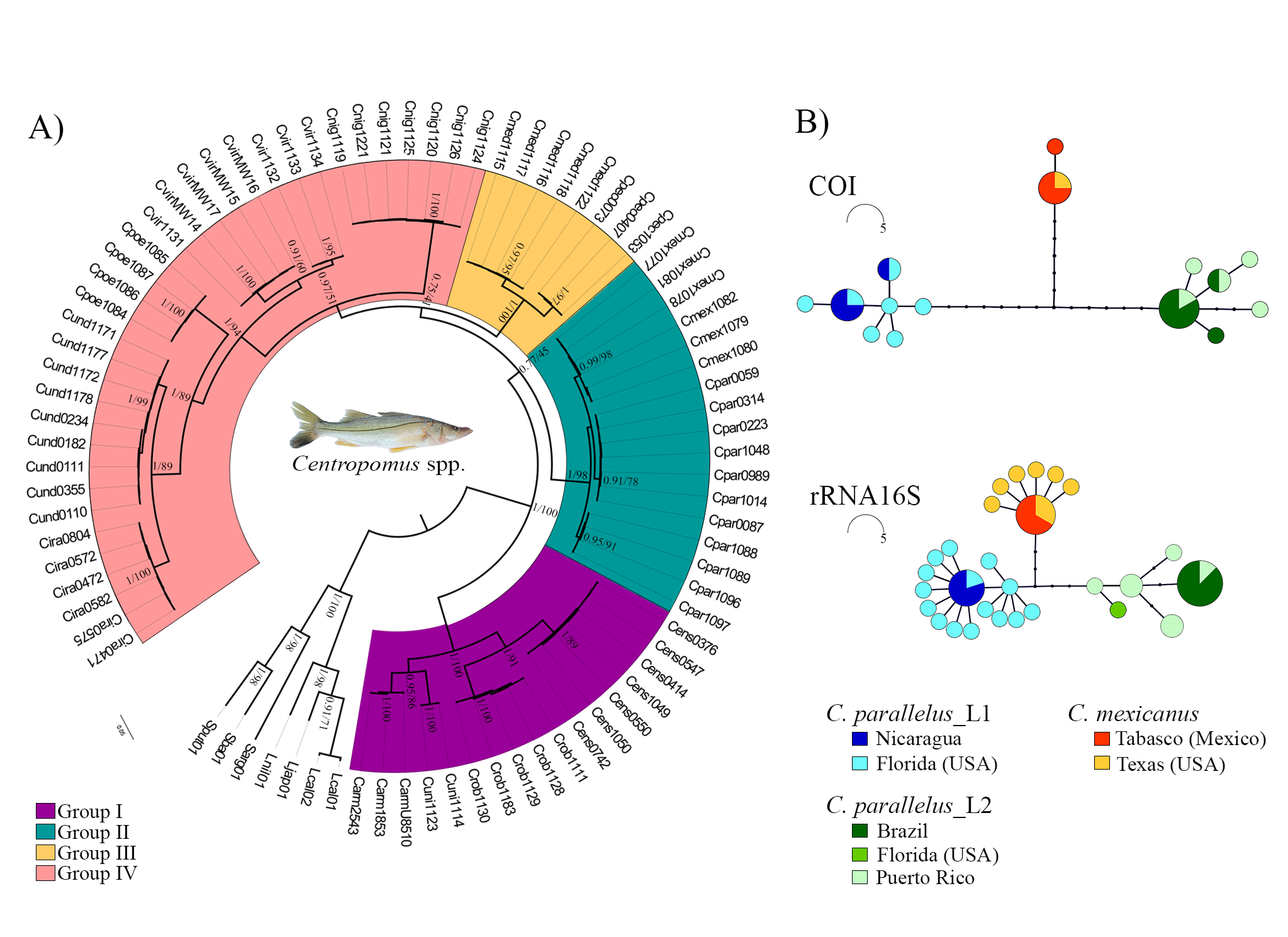

Supplement: S1 Fig — B) the mitochondrial haplotype network of the C. parallelus/C. mexicanus complex. In the mtDNA topology, the values shown in the nodes indicate the statistical support defined by the Bayesian Posterior Probability (BPP) and Bootstrap (BS) values, respectively. Centropomus irae obtained by the authors represents the genus. In the haplotype network the scale (size of the circle) is proportional to the number of individuals with the respective haplotype. (TIF) [file pone.0332412.s005.tif]
